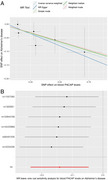# Higher blood pituitary adenylate cyclase‐activating polypeptide levels were causally associated with a decreased risk of Alzheimer's disease

**DOI:** 10.1002/alz70856_096870

**Published:** 2025-12-24

**Authors:** Xingzhi Guo, Rui Li

**Affiliations:** ^1^ Shaanxi Provincial People's Hospital, Xi’an, Shaanxi Provincial, China

## Abstract

**Background:**

Previous studies in humans and animals have suggested that pituitary adenylate cyclase‐activating polypeptide (PACAP) is associated with Alzheimer's disease (AD) pathology. However, whether blood PACAP levels are causally linked to AD susceptibility remains largely unknown.

**Method:**

A two‐sample Mendelian randomization (MR) analysis was performed to assess the causal effect of blood PACAP levels on the risk of AD using genome‐wide association studies (GWAS) summary statistics. The instrumental variable (IV, *p* <5E‐06, LD: r^2^<0.001 in 10000kb) for blood PACAP was selected from summary‐level data based on blood plasma proteome GWAS of European descent (*N* = 3,301) [1]. For the outcome, GWAS summary statistics on AD were obtained from the International Genomics of Alzheimer's Project (IGAP, *N* = 63,926) [2]. Five different statistical methods, including inverse‐variance weighted (IVW), MR‐Egger, weighted median, simple mode, and weighted mode, were applied to calculate the MR estimate. Meanwhile, sensitivity analyses, including pleiotropy and heterogeneity tests, were also conducted to evaluate the stability of the results.

**Result:**

MR estimates based on the IVW method showed that genetically proxied higher blood PACAP levels were causally linked to a decreased risk of AD (OR=0.87, 95%CI=0.79‐0.95, *p* = 0.003), which was confirmed by other MR approaches (Figure 1A). No significant horizontal pleiotropy and heterogeneity were observed in sensitivity analyses, indicating good robustness of the findings. Additionally, the leave‐one‐out analysis indicated no single instrumental variable, which led to biased results (Figure 1B).

**Conclusion:**

The present MR study demonstrates that blood PACAP levels causally contribute to lower susceptibility to AD, suggesting that PACAP might serve as a potential target for diagnosis or therapy of AD.